# Experiences, impacts, and requirements of synchronous video consultations between nurses, allied health professionals, psychological therapists, and adult service-users: A review of the literature

**DOI:** 10.1371/journal.pdig.0000654

**Published:** 2024-12-04

**Authors:** Lynn Mcvey, Martin Fitzgerald, Jane Montague, Claire Sutton, Peter Branney, Amanda Briggs, Michael Chater, Lisa Edwards, Emma Eyers, Karen Khan, Zaid Olayiwola Olanrewaju, Rebecca Randell

**Affiliations:** 1 Faculty of Health Studies, University of Bradford, Bradford, West Yorkshire, United Kingdom; 2 Wolfson Centre for Applied Research, West Yorkshire, United Kingdom; 3 Centre for Digital Innovations in Health & Social Care, University of Bradford, Bradford, West Yorkshire, United Kingdom; Iran University of Medical Sciences, ISLAMIC REPUBLIC OF IRAN

## Abstract

**Background:**

Telemedicine is increasingly used within healthcare worldwide. More is known about its efficacy in treating different conditions and its application to different contexts than about service-users’ and practitioners’ experiences or how best to support implementation.

**Aims:**

To review adult service-users’ experiences of synchronous video consultations with nurses, allied health professionals and psychological therapists, find out how consultations impact different groups of service-users and identify requirements for their conduct at individual, organisational, regional, and national levels.

**Method:**

CINAHL, Embase, Medline, PsycINFO Scopus were searched for papers published between 01/01/2018 and 19/03/2021. One reviewer independently reviewed citations and a second reviewed those excluded by the first, in a liberal accelerated approach. Quality assessment was undertaken using the Mixed Methods Appraisal Tool and data were synthesised narratively.

**Results:**

65 papers were included. Service-users’ experiences of video consultations ranged from feelings of connection to disconnection and ease of access to challenges to access. Many were excluded from video consultation services or research, for example because of lack of access to technology. Individual service-users required clear orientation and ongoing technical support, whereas staff needed support to develop technical and online-relational skills. At organisational levels, technology needed to be made available to users through equipment loan or service models such as hub-and-spoke; services required careful planning and integration within organisational systems; and security needed to be assured. Regional and national requirements related to interorganisational cooperation and developing functionality.

**Conclusion:**

To support safe and equitable video consultation provision, we recommend: (1) providers and researchers consider how to maximise participation, for example through inclusive consent processes and eligibility criteria; (2) sharing video consultation user guides and technical support documentation; and (3) continuing professional development for practitioners, focusing on the technical and relational skills that service-users value, such as the ability to convey empathy online.

## Introduction

Telehealth is defined by the World Health Organisation as the use of electronic communications and information technologies to support healthcare services, while telemedicine uses information and communication technologies to exchange information for the diagnosis, treatment and prevention of disease and injuries [1]. Such approaches have been used since the second half of the 20th century, particularly to treat chronically ill patients who find it difficult to leave their homes and those living in remote areas [2–4]. Since the COVID-19 pandemic, use has grown rapidly worldwide [5,6]. Reflecting this, in 2022 it was estimated that global telehealth markets would rise at a compound annual growth rate of 26.6% to £285.7bn by 2027 [7].

*Telemedicine has been shown to be equally or more effective than other delivery modes in certain circumstances and for certain conditions [3,8]. For example, a Cochrane review found no difference in mortality between patients with heart failure receiving care through interactive telemedicine, compared to those receiving care without telemedicine [9]. But there are also challenges, including adjusting to differences in conversational cues online [10], technical problems [3], and issues of digital exclusion [2]. As telemedicine becomes more common, there is a pressing need to understand its impact on services and service-users. Existing reviews often focus on the effectiveness of telehealth interventions in treating different conditions [8,9,11]; its application in different types of provision, such as primary or critical care [12,13]; or on the use of technology [3]. Less is known about service-users’ experiences of this delivery mode, nor those of the varied practitioners who offer it [14], and there is also a need to better understand and support implementation [15,16]*. *Review questions*

Given this background, this review seeks to answer the following questions:

1. What are adult service-users’ experiences of synchronous video consultations (VCs)—appointments that take place in real time between a patient and a healthcare professional over video, as opposed to face-to-face—with nurses, midwives, allied health professionals, and/or psychological therapists?
2. Do VCs impact in specific ways on groups of service-users?
3. What are the micro- (individual), meso- (organisational), and macro-level (interorganisational) requirements for the conduct of synchronous VCs?

VCs were selected from among different types of telemedicine because we believed this developing technology, with its audio-visual elements, was likely to elicit rich experiential data. VCs with nurses, midwives, allied health professionals, and psychological therapists were reviewed. Given the existing emphasis in research and reviews on the effectiveness of telehealth for different conditions, much is known already about consultations offered by doctors, so we did not include them. Psychological therapists were included owing to their long-standing involvement in remote consultations [4,17] and relational expertise, which, we hypothesised, might offer the potential for inter-professional learning. The review also responds to calls for research that supports implementation by examining practical requirements for teleconsultation conduct.

## Methods

This unfunded review was carried out by the University of Bradford School of Health Studies’ Technologies for Quality and Safety Group and Allied Health Professions University and Clinical Partners Working Group on Telehealth, with support and advice from service-users and carers. The review was led by a Research Fellow in applied health research (LM), with experience of research into the use of technology in healthcare and of working as a psychotherapist remotely. The 11 colleagues who also participated included a Professor of Digital Innovations in Healthcare; staff with teaching/research roles in undergraduate and postgraduate health-related programmes in midwifery, nursing, occupational therapy, physiotherapy and paramedic science; and doctoral researchers.

A rapid review method was followed [18], which streamlines the process of conducting a systematic review [19]. This involved taking a liberal accelerated approach to screening abstracts and texts [20], where a proportion of abstracts and texts are dual-screened to test reviewer concordance, after which one reviewer screens the remaining materials and a second screens those excluded by the first reviewer. This is intended to be less time and resource-intensive than 2 reviewers reviewing all citations independently, while maximising inclusion and increasing numbers of retained texts. The method was chosen owing to its robustness and efficiency. We wanted to produce high-quality evidence in a resource-efficient manner, enabling reviewers with existing work commitments to contribute.

An information specialist was consulted during the development of the search strategy and conducted searches (see Tables 1 and 2).

### Protocol registration

The review was registered on the PROSPERO International Prospective Register of Systematic reviews protocol (CRD42021250932) [21].

### Inclusion and exclusion criteria

**Table 1:** Inclusion and exclusion criteria.

	Inclusion criteria		Exclusion criteria
PICO criteria			
Population	Service Users: Adults aged over 18 years participating in synchronous VCs for their own health or the health of an adult third-party. Practitioners: Nurses, midwives, consulting allied health professionals, and psychological therapists		
Intervention	Therapeutic or rehabilitative Video-conferencing technology to establish real-time, two-way audio-visual links between service-users and practitioners, one-to-one or in groups		Telephone consultations Asynchronous consultations VCs solely between practitioners
Comparator	Where available, comparisons were made between VCs and face-to-face consultations		
Outcome	All process and outcome measures of synchronous VCs were included		
Search strategy			
Study type	English language		Non-English Abstracts, opinion pieces, editorials, conference abstracts, books, book chapters, PhD theses
Time limit	2018 onwards (due to rapidly evolving technology)		

### Search strategy and article selection

**Table 2:** Search strategy.

Database	CINAHL, Cochrane CENTRAL, Embase, Medline, PsycINFO, Scopus (Title, abstract)
Date range	01/01/2018 to 19/03/2021
Language	English
Strategy	#1 AND #2 AND #3
#1	“Digitally enabled service*” OR “digital health” OR “e-care” OR econsult* OR “e-consult*” OR “e-diagnos*” OR ediagnos* OR ehealth OR “e-health” OR emedicine OR “e-medicine” OR enurs* OR “e-nurs*” OR epsych* OR “e-psych*” OR etherap* OR “e-therap*” OR “internet based intervention” OR “non face-to-face clinic*” OR “non-face-to-face clinic*” OR “non face to face clinic*” OR “remote consult*” OR “remote prescri*” OR “rural consultat*” OR “tele-based” OR telebased OR telecare OR “tele-care” OR “tele-communic*” OR telecommunic* OR teleconsult* OR “tele-consult*” OR telecounsel* OR “tele-counsel*” OR telehealth OR “tele health” OR teleintervention* OR “tele-intervention*” OR telemedicine OR “tele-medicine” OR telemonitor* OR “tele-monitor*” OR telerehab* OR telenurs* OR “tele-nurs*” OR “tele-rehab*” OR telepatholog* OR “tele-patholog*” OR telepsych* OR “tele-psych*” OR teletherap* OR “tele-therap*” OR teletreat* OR “tele-treat*” OR “video consult*” OR “video-consult*” OR videoconferenc*OR “video-conferenc*” OR “virtual clinic*” OR “virtual working”
#2	“Advanced practi*” OR “allied health profession*” OR “biomedical scientist*” OR chiropodist* OR “clinical scientist*” OR “cognitive behavio* therap*” OR counsel* OR dietician* OR dietitian* OR “hearing aid dispens*” OR midwi* OR nurs* OR “operating department practitioner*” OR osteopath* OR orthopist* OR orthotist* OR paramedic* OR physiotherap* OR podiatrist* OR prosthetist* OR psycholog* OR psychotherap* OR radiograph* OR therap*
#3	Appropriate OR dissatisf* OR exclud* OR experienc* OR fail* OR happ* OR impact* OR inappropriate OR include* OR need* OR opinion* OR outcome* OR process* OR “patient reported outcome measure*” OR “patient-reported outcome measure*” OR PROM* OR “patient reported experience measure*” OR “patient-reported experience measure*” OR “PREM*” OR require* OR satisf* OR successful* OR support* OR understand* OR unhapp* OR unsatisf* OR unsuccessful* OR "work* well"

### Data extraction

Retrieved records were imported into EndNote, where duplicates were removed. Several reviewers screened abstracts and full texts independently (LM, JM, LE, EE, ZO, MC, MF, CS, KK, AB, PB), using the liberal accelerated approach outlined above.

Data were extracted independently from the included papers by LM, PB, JM, LE, EE, ZO, MC, MF, and CS using a form developed by and piloted among the review team. Second reviewers extracted data from a 10% sample of manuscripts, to check for correctness and completeness of extracted data. The review lead checked all extracted data. The information extracted for each study, together with a brief explanation of what each heading included, is summarised in **Table 3**, while an overview of extracted data is provided in **S1 Data.** We collected information relating to the headings in Table 3 and found no missing or unclear information.

**Table 3:** Summary of data types extracted.

Heading	Description
Reference	An abbreviated reference for the study, listing the first author’s surname and title of the text.
Year of publication	Year of publication of the text.
Country	Country or countries in which the study took place.
Study design	Specifies whether the study used quantitative, qualitative or mixed methods, and provides a brief summary of research design.
Sample type and size	Briefly describes sample size and type of sample, e.g., random, cluster, purposive, snowball.
Setting	The setting or context in which the study was carried out, such as in a hospital or a community venue.
Professionals involved	Lists the professionals involved in the study from among the groups included in this review, namely nurses, midwives, allied health professionals, and/or psychological therapists.
Nature of intervention	Summarises intervention(s) reported by the study, including type of consultation and functionality provided by software.
Control or comparator	Lists any controls or comparators used, where applicable.
Any reported process and outcome measures	Summarises process or outcome measures reported in the study, such as questionnaires or inventories.
Was video consultation as effective as other forms of treatment?	Summarises information in the text (if given) about whether VCs were as effective as other forms of treatment. This category applied to studies that compared VCs with other modalities.
Summarise what the paper says about adults’ experiences of synchronous video consultations	A summary of information in the text about adults’ experiences of VCs with included professionals. May include relationship/emotional connection established between patient and practitioner during the consultation, and what supported or constrained this. This information relates to research question 1 in this review, which asks “What are adult service-users’ experiences of synchronous VCs?”
Where reported, summarise characteristics of those whose experiences are reported in the paper, particularly UK protected characteristics.	A summary of service-user characteristics, including (where noted in the text) characteristics protected in UK law against discrimination: age, disability, gender reassignment, marriage and civil partnership, pregnancy and maternity, race/ethnicity, religion or belief, sex and/or sexual orientation. Also includes migration status. This information relates to research question 2 in this review, which asks “Do VCs impact in specific ways on groups of service-users?”
Summarise what the paper says about micro-level requirements for conducting and supporting synchronous video consultations	A summary of micro-level requirements identified or implied by authors for conducting and supporting VCs, such as access to the internet and availability of appropriate equipment for patients, carers, and professionals, or requirements in conducting interpersonal interactions. This information relates to research question 3 in this review, which asks about requirements for the conduct of synchronous VCs.
Summarise what the paper says about meso-level requirements for conducting and supporting synchronous video consultations	Summary of meso- or organisational-level requirements identified or implied by authors for conducting and supporting VCs, relating to research question 3 in this review.
Summarise what the paper says about macro-level requirements for conducting and supporting synchronous video consultations	Summary of macro-level requirements identified or implied by authors for conducting and supporting VCs, relating to research question 3 in this review. These requirements could apply at interorganisational, regional, national, or international levels.

### Quality assessment

The Mixed Methods Appraisal Tool (MMAT) was used to assess quality, see **S1 File** [22]. The MMAT is a validated tool for appraising the methodological quality of quantitative, qualitative, and mixed methods study designs. For each study, the tool requires reviewers to indicate whether there were clear research questions, and whether the collected data allowed these questions to be addressed, before then appraising the specific design, using criteria appropriate to quantitative, qualitative, and mixed methods studies. For example, for qualitative studies, reviewers must indicate whether there is coherence between qualitative data sources, collection, analysis, and interpretation. A yes/no answer is provided for each criterion, the combination of which can be used to derive a quality rating for each study. We used a “star” system to represent the quality rating for each paper in the MMAT Table, with 5 stars indicating that 100% of quality criteria were considered to be met; 4 stars that 80% quality criteria were met; 3 stars that 60% were met; 2 stars that 40% were met; and 1 start that 20% were met. A single reviewer independently rated each paper, with verification of a 10% sample of judgements by a second reviewer.

### Data synthesis

The data were synthesised narratively, structured around the review questions. The synthesis was carried out by the review lead, who read through the included papers and their summaries in the Study Details Table (see **S1 Data**), before integrating findings in themes linked to the research questions for the review. A draft report of the themes and categorised findings was circulated to the wider review group for comments, which were taken into account before being re-circulated for further comment and refinement.

## Results

### Characteristics of included studies

**Fig 1:**
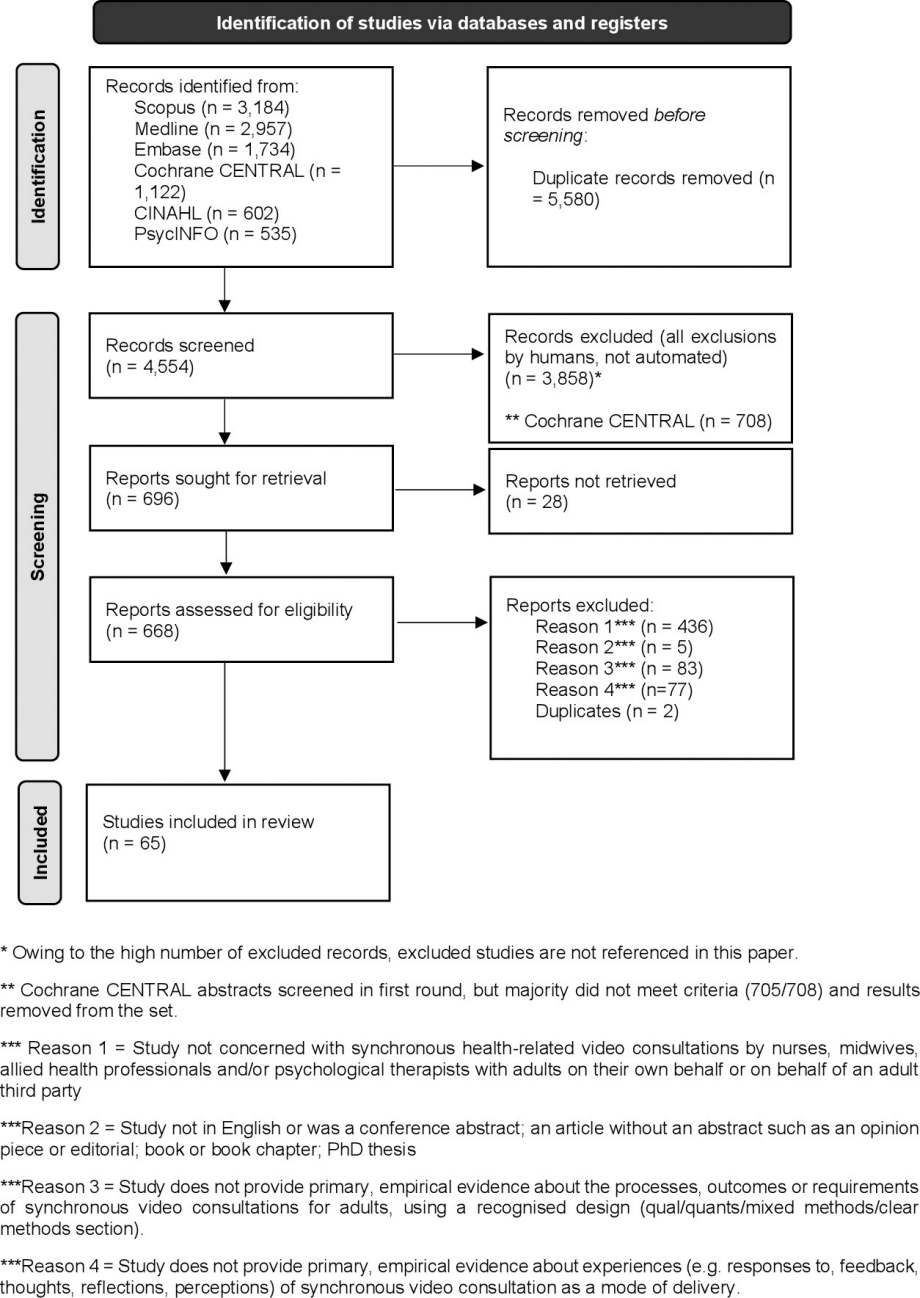
PRISMA diagram about here.

Sixty-five papers were included: see **Fig 1** [23–87]. In 54 (83%) at least 60% of quality criteria were met: details are provided in **S1 File**. Generally speaking, the quality of quantitative and qualitative papers included in the review was good, but the quality of mixed methods studies was more variable, because some studies, while using both quantitative and qualitative methods, did not frame themselves as mixed methods, gave no rationale for the design used and did not explain how data were analysed, making it difficult to ascertain whether the potential for bias had been mitigated. Forty two per cent of the papers had a mixed methods design, 35% were quantitative, and 23% qualitative. Studies were primarily carried out in Western nations, the top 3 being the United States of America [23,24,27,30,41,43–45,48,50,51,54,55,61,66,71,72,75,77,79,82,83,86,87], Australia [29,47,57,59,62,65,69,73,74,76,80,84,85], and the United Kingdom [25,33,39,40,49,64,67,81]; see **S1 Data.** A diverse range of service-users participated. The most frequently reported characteristics were age, sex, race/ethnicity, and marital status, with diversity in these groups across samples. VCs were most often offered in the included papers by psychological therapists, who featured in 28 studies [24,25,32–36,38,42,44–46,50,51,54,55,57,58,64,66,67,69,70,75,79,82,83,86]. The next most populous group was nurses in 17 studies [23,26–28,38,41,46,48,49,53,56,60–62,66,77,81], followed by physiotherapists/exercise physiologists/physical therapists in 15 studies [29,30,40,43,47,52,60,62,63,65,71,72,80,84,85]. Dieticians [29,30,39,59,80,84,85] and speech language therapists or pathologists [29,30,39,59,80,84,85] were each involved in 7 studies, and occupational therapists in 4 [31,40,71,87]. In several studies more than 1 practitioner group offered a service [29,30,38,40,46,60,62,66,71,80,84,85]. None of the papers featured midwives.

Where studies included comparisons such as with face-to-face provision, VCs were found to be comparable or better [24,26,33–35,45,48,52,54,65,69,71,75,80] with some exceptions [23,36,53]. Akhtar and colleagues [23] compared VC and in-person examination of patients presenting to an emergency department with a sore throat and found VCs performed poorly for detecting abnormal submandibular lymph nodes or asymmetry of the posterior pharynx. Carotenuto, Rea [36] examined the assessment of patients with Alzheimer’s disease via VC and face-to-face and found VCs overestimated the cognitive impairment of patients with more pronounced cognitive deficits. The authors speculate that this was probably due to patients’ difficulties in understanding the meaning of specific questions in the assessment, although they do not explain why this was more difficult online than face-to-face. Kazawa, Osaki [53] compared the efficacy of nurse-led VCs and face-to-face interviews for promoting behavioural change and disease management in patients with diabetic nephropathy and found greater improvement in behaviour changes regarding self-monitoring in the face-to-face group.

### Summary of results

The following narrative synthesis of findings is structured in relation to the review’s 3 review questions: experiences of VCs; impact on service users; and requirements for VCs. In terms of experience, some service users reported feeling empowered and enabled by VCs; conversely, others felt disconnected and disempowered. There were also differences in access to VCs, with some users valuing their flexibility and others reporting difficulties, including technical problems.

Positive impacts were reported across the age spectrum although some older users found them challenging and preferred face-to-face consultations. Impacts could also vary according to the nature of users’ mental health issues and VCs were found to be unsuitable for people with some mental health problems. Likewise, impact was variable for users with poor physical health, with some conditions requiring hands-on treatment. Some users were excluded from studies, owing to their language skills or lack of access to technology, for example.

**Fig 2:**
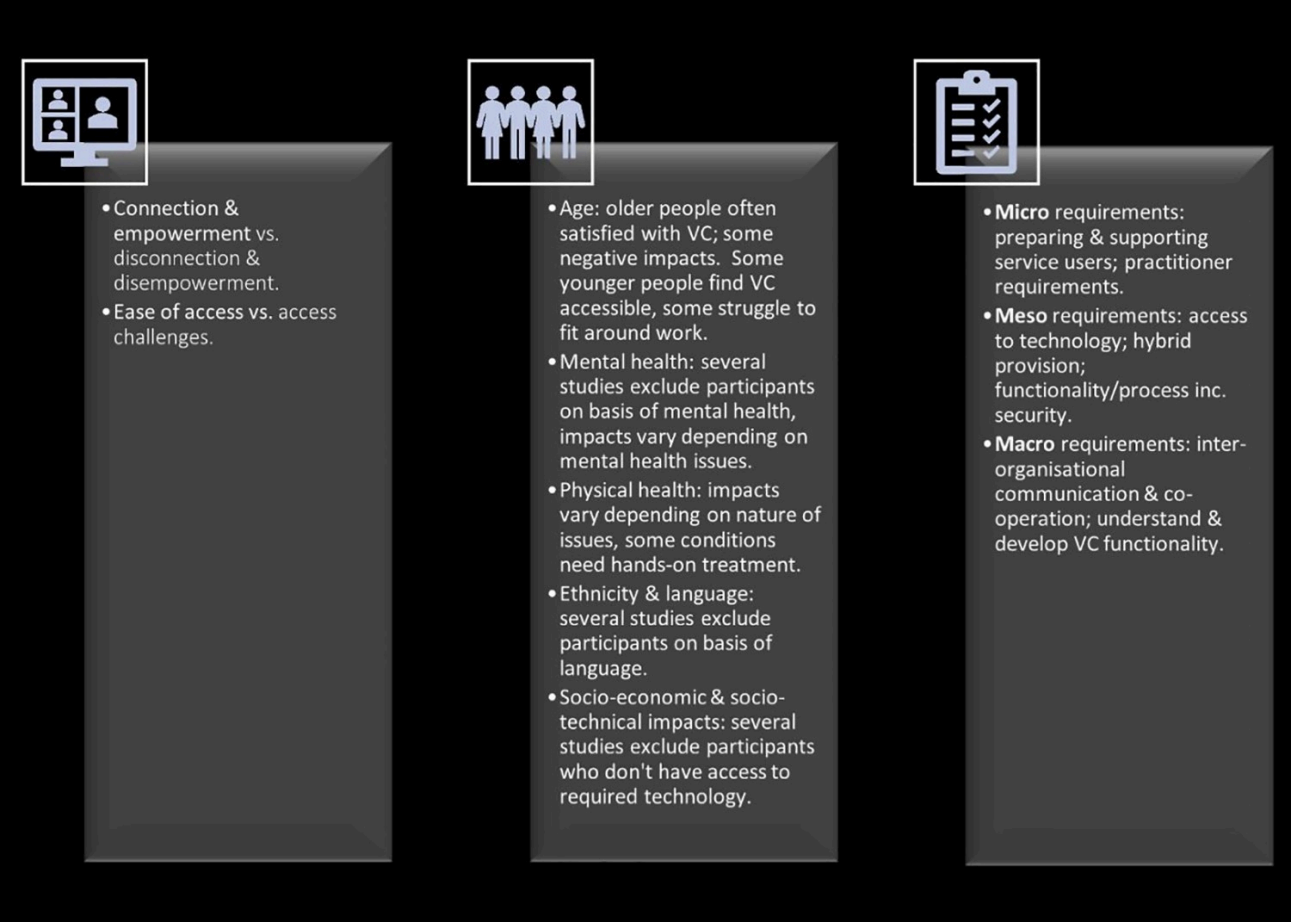
Theme summary about here.

The review found that for VCs to be effective at micro-levels, service users needed to be supported (e.g., with technical advice) and practitioners aware of the requirements of the consultation. At meso-levels, access to technology was required, and hybrid provision (which included some face-to-face contact) was desirable. There was a need to be cognisant of issues of security, including functionality and process. Macro requirements included effective communication and cooperation between organisations as well as understanding functionality. See also **Fig 2** below.

### Experiences of synchronous video consultations (RQ1): From feelings of connection and empowerment to disconnection or disempowerment

For some patients, VCs offered a sense of personal connection or empowerment compared to other delivery modes. Examples include providing a sense of ease in one’s own environment, compared to organisational accommodation [25,39,75,78]; feeling less confrontational than face-to-face appointments [29,33,44]; or reducing the experience of stigma or discrimination of face-to-face appointments when attending a weight management programme [39] or abortion information visits [41]. Some aspects of videoconferencing technology were thought to enhance these feelings of engagement and emotional connection, such as the use of the chat function in Microsoft Teams software as an additional means of group interaction in mindfulness-based art therapy for people with multiple sclerosis [66] or the use of text, pointer and drawing functions in Adobe Connect to promote interactivity in a telerehabilitation group for patients with aphasia [74].

Studies involving psychological therapists and mental health practitioners provided some evidence about how these feelings of connection were generated. Norwood and colleagues [67] for example, examined the working alliance (the collaboration between service-users and practitioners in terms of goals, tasks, and the bond between the pair) between therapists and service-users in a study of CBT for health anxiety delivered via VC. Where service-users developed a positive alliance with their therapists, this was often attributed to their relational and therapeutic skills and attitudes, such as the ability to be open and honest; allowing service-users time to talk, while also bringing them back to topic when needed; and developing shared understandings. Other studies provided evidence that psychological therapists could work with the physical distance involved in VCs to help service-users take greater ownership of the therapeutic change process. For example, in a study of couples counselling, some participants talked about being less likely to rely on the therapist to make changes for them, because the therapist was not in the same room [57]: “*because you know there’s the two of us in this room… we’re the ones doing it*, *you know ‘A’ [therapist] is more kind of guiding us as opposed to if she was in the room we might feel a little bit more like we couldn’t do it on our own*.” (p. 10). (See also “Micro requirements” below, which outlines the qualities, attitudes and skills practitioners needed to connect emotionally with patients or clients while working online.)

In other cases, however, both service-users and practitioners experienced a lack of connection associated with aspects of VCs. Some perceived them to be more impersonal than face-to-face consultations [25,27,32,40,44,57,59]. A participant in a telerehabilitation intervention for people with dementia put it like this: “*I do miss the face-to-face*. *I just think it’s having somebody here with me*, *I can’t really explain it*, *it just doesn’t feel the same*” [40] (p. 6). Although service-users sometimes valued the sense of distance from practitioners in VCs and thought it helped them to be more open in treatment [39,44,67,78], others felt less able to trust practitioners [42,57]. For some, it was easier to avoid engaging with challenging material in VCs than when attending in-person meetings because they could switch off (literally and figuratively) [25], while for others, disengagement was more logistical, with service-users reporting more difficulty remembering to attend remote sessions than in-person participants (difference = 0.23, 0.04) [54].

Disruption to the social processes that occur naturally in face-to-face interactions was also reported, caused or exacerbated by technical issues such as unstable network connections [42,53]. Participants reported difficulties perceiving non-verbal communication such as body language or facial expression [25,38,53,78]. As a result, people could miss or misinterpret relational information. Rietdijk and colleagues [78] identify a risk of clinicians not noticing fidgeting movements as participants’ attention faded and therefore not responding accordingly. A further difficulty related to the abruptness with which sessions could end. This could leave service-users having to process feelings on their own, especially following emotionally sensitive interactions [25,33,64]. A service-user in a cognitive processing therapy intervention for veterans with post-traumatic stress disorder described this: “*Then you’re kind of instantly back into the room as it were and whatever your mind and emotions are in the course of that hour*, *suddenly that therapeutic bubble is burst and you’re back to being with all the normal stuff around you … it’s a very jarring*, *very sudden transition*” [25] (p. 5). Finally, some service-users did not feel comfortable attending consultations in their own homes because they had no private space [25,44]; might be disturbed [25,70]; or felt the consultation to be intrusive [67].

### Experiences of synchronous video consultations (RQ1): From ease of access to access challenges

Many service-users appreciated the ease of access to consultations offered by VCs, owing to not travelling, easier integration with other commitments (including work and care responsibilities), and reduced costs and time [25,27–29,32,33,37–42,44,47,49,54,56,60,61,64,65,67–69,72,73,76,78–80,85,87]. A veteran soldier participating in CBT for suicide prevention summarised his experience as follows: “*It was much more convenient; my commute could be over an hour and a half; I’d arrive to the office agitated by traffic; it’s more relaxing*” [79] (p. 5). The accessibility offered by VCs was the determining factor in whether some service-users were able to engage with treatment at all [39,42,44,64,67]. Several papers also reported that service-users found VCs easy to use [37,41,47,49,55,62,65,68,74,84–86].

Conversely, there were frequent challenges to accessing VCs, associated with technical problems such as poor internet connectivity [25,27,31–33,37–39,44,47,55,66,68,70,73,74,76–78,82]; poor sound and image quality [25,41,53,55,57,60,63,68,73,74,78,79,86]; difficulty logging in or remaining connected [77,85]; and difficulty installing software/hardware, software incompatibility or glitches [39.80]. Service-users sometimes struggled to use features such as screen-sharing or link-sharing functions [78]. A study in which patients with heart failure carried out self-examination in VCs with specialist nurses suggests that service-user competence with equipment and technology could impact on their care [81]. Here, the authors provide an example of a patient taking an inaccurate oxygen saturation reading which the nurse accepted until, by chance, the inaccuracy was revealed.

These technical issues could impact on both service-users’ and practitioners’ sense of connection and empowerment in VC, by, for example, interrupting conversational flow [27,73,74] leading “*to both patient and provider frustration and internalized feelings of incompetence with the technology*” (p. 431) [38]. However, as familiarity with use improved, so too did user appraisal, with overall positive acceptance and valuing of VC technology and an understanding that glitches are a reality of such provision [33,57,73,74].

### Impacts on groups of service-users (RQ2)

#### Age-related impacts

Several studies showed older service-users valuing VCs and finding the technology accessible [38,64,68,75,83,87]. In their trial comparing VCs and in-person delivery of behavioural activation therapy for depression to veterans, Pruitt and colleagues [75] found greater end-of-treatment satisfaction for VCs was more commonly associated with older, more senior and symptomatic veterans, compared to younger counterparts. Other studies found no relation between age and variables such as treatment completion [64] or occurrence of technical faults [68].

In other cases, elements of VCs impacted older patients negatively, often linked to their mental or physical health [27,32,48,65,78]. In one study, older veterans with complex medical conditions and suspected mild cognitive impairment and their carers communicated with nurses via VCs on iPads or tablets, but concerns were expressed that frail patients might fall when walking with devices to show nurses their living spaces [48]. Although most of the older service-users reported some positive aspects of VCs, less than half wanted to see their healthcare provider by VC in the future [48].

Younger people could also be impacted by aspects of VCs. While VCs are reported above as making it easier for people to meet work-life commitments by removing the need for travel, experiences were not always positive. Barnett and colleagues [29] reported that, in a 12-week group lifestyle and exercise programme for liver transplant recipients, participants in employment consistently prioritised work above telehealth appointments and could feel too tired to engage in the exercise component of the programme after work hours.

#### Mental health-related impacts

Over half the studies in this review excluded participants owing to aspects of their mental health [23–27,30–33,35,36,38,42,44–48,50,53–58,60,62,64–66,68–70,79,85–87]. In studies in which psychological therapies were offered, this was often done either to avoid confounding results (as, for example, in a study about psychological treatment for patients with chronic pain, which excluded participants receiving similar treatment for any other disorder [58] or for clinical reasons. Certain therapies are contraindicated for some mental health conditions, whatever the delivery method. For example, in a randomised controlled trial of cognitive behavioural therapy (CBT) for insomnia, exclusion criteria included presence of Axis I psychiatric disorders for which CBT for insomnia may be contraindicated (e.g., bipolar disorder, psychotic disorder, and active substance use disorder) [24]. Where a therapy was specifically designed to address acute mental health conditions such as suicidality, there was some evidence that it could be offered safely via VC, with careful management. In a paper involving CBT via VC for suicide prevention, Rojas and colleagues [79] reported measures taken to safeguard a recently discharged veteran with suicidal ideation. Safeguards included the veteran agreeing to stay connected by video during a clinical emergency; if video connection was lost, agreeing to reconnect or wait for the therapist to reconnect by phone; providing a local emergency contact and agreeing to the therapist using emergency services if needed. This was, however, a single-case report.

In other cases, people were excluded from VC because they did not have mental capacity to consent to the research or, in researchers’ or clinicians’ judgement, to participate in VC [32,44,46,50,60,69,70,87]. In a study about occupational telehealth [87], for example, exclusion criteria included a diagnosis of dementia or moderate to severe cognitive deficits that “*would impair ability to provide informed consent*” (p. 106) and participants needed “*cognitive skills permitting use of telehealth technology*” (*Ibid*.). Impacts of VCs on service-users could vary according to the nature of their mental health issues. Service-users undertaking CBT for health anxiety liked VC’s “*environmental freedom*” (p. 1340) and felt more comfortable in their own surroundings [67], although one felt intruded on by the video-link to their home: “*Whenever I video conferenced in my bedroom* [the therapist] *could see that I’m still definitely very obsessive about being tidy*” (p. 1340). In a study of teletherapy for binge-eating disorder, it was found that patients who are anxious and/or avoidant may be more willing to attend VCs because they experience them as less confronting than face-to-face sessions [44]. The authors noted that it is not yet known whether the facility to see oneself on screen during VCs may increase self-focus or social comparisons during therapy for this group and recommended further research.

VCs were found to be unsuitable for people with some mental health problems. For example, both patients and providers in a study about treatment for older people with depression agreed that VCs were more appropriate for patients with mild to moderate depression and as a supportive technology. They believed it would be harder to establish an effective therapeutic relationship via VC in more severe cases [38].

#### Physical health-related impacts

VCs were used to provide treatment to people who struggled to attend in-person appointments owing to mobility restrictions or frailty; people who needed many treatment sessions or lived in rural settings [28,33,42,49,77]; or who had infectious conditions [63]. Some physical health conditions were difficult to manage through VCs because they needed hands-on assessment or therapy, such as Achilles tendinopathy [47] and stoma care [49]. In a nurse-led programme for patients with diabetic nephropathy, some service-users struggled to see images or the nurse when using the tablet [53].

As with mental health, service-users were sometimes excluded from studies owing to their physical health, often because a service was designed to meet the needs of a particular group of patients or to exclude confounding conditions. In a study of voice therapy for Parkinson’s disease people were excluded if they had a known history of neurological disease other than Parkinson’s or of a speech disorder [37]. Service-users were also excluded from some studies because researchers or clinicians judged that a physical impairment would make it difficult for them to participate, as in an occupational therapy intervention, which required “*good to adequate fine motor dexterity to operate electronic device*” (p. 106) [87].

#### Language-related impacts

Many studies excluded participants if they could not speak or communicate in the main language(s) of the country in which the research was taking place [23,27,29–33,40,41,47,51,56,60,61,66,69–72,77–79,85,87]. Little information was given in the included papers about impacts on people in terms of their language, although in one weight management programme a service-user with English as a second language talked about the added difficulty of understanding others when there were technical issues: *“Because English is my second language*, *I need to pay more attention when other people are talking so*, *dropping and freezing and this type of thing were distracting*, *but not on an extent of spoiling the experience*” (p. 71) [39].

#### Socioeconomic and -technical impacts

Service-users were excluded from several studies if they did not have internet access/WiFi at home or in a space of their choosing [31,33,35,38–40,43,46,56,64,69,70,72,76,78,80,84,85,87] or a computer, smart-phone, or other equipment such as a built-in camera with which to engage in VC [33,35,38–40,43,49,56,61,69,72,76,78,85,87]. Such criteria may have reduced the availability of VCs for people from lower socioeconomic backgrounds and people living in areas with limited internet cover, such as rural regions [42,61,77]. However, as a practitioner pointed out in a VC programme for patients with persistent asthma, such service-users might be among those most in need of VCs: “*The population we work with transportation is a big limiting factor*, *they have to take off work and they can’t afford to miss a day’s pay*, *or they have children they need child care for*, *so they miss their appointments*” [61] (p. 7).

### Requirements for conducting and supporting synchronous video consultations (RQ3)

#### Micro requirements

Preparatory information and support (test calls, set-up meetings, user guides) helped service-users understand and use VCs [30,36–40,42,43,45,47,48,62,64,65,69,71,76,77,79,87]. In some cases, ongoing technical support was provided to service-users [27,31,36,42,50,60,62,63,68,86], either through practitioners or organisational teams. Such support may therefore be regarded as a requirement for some service-users to access VCs. Within this, authors drew attention to the utility of remote access software—which enables remote control of a device—to help manage technical issues, because technicians can address issues on service-users’ computer themselves [62,63,68]. For example, in a VC speech therapy intervention for patients with aphasia following a stroke, speech pathologists used the software LogMeIn on study laptops loaned to participants, which enabled remote control if needed [68]. They acknowledged the importance of this facility for this patient group and recommended such software in VCs with patients with cognitive or communication impairments. However, they also pointed to problems if participants used their own devices, because the software may enable access to private material.

Requirements for individual practitioners included having or developing sufficient technical skills to deliver VCs and support participants (especially if no organisational technical support was provided). Some authors recommended technical training for practitioners [32,37,47,68,72,73]. In an aphasia group therapy programme, speech language pathologists were trained for 1 to 2 h in use of the VC platform, covering functionality and providing opportunities to practice skills through role-play [73].

Practitioners also needed certain qualities, attitudes, and skills to build and sustain trusting therapeutic relationships online and generate the feelings of connection and empowerment service-users valued. Patients said practitioners needed to be able to relate to them in trustworthy ways, to be empathic and take them seriously [32]. Some papers reported that trust was generated when practitioners discussed “ground rules” such as how confidentiality would be assured [39,44,67]. Others pointed to skills needed to maintain focused attention on service-users [28, 61], for example, by looking (or appearing to look) directly at them through video cameras [61]. Practitioners also needed to help service-users focus on consultations. In a couples counselling intervention, VC was set up so the couple could see only the therapist (and not themselves), to reduce distraction [57]. To enhance service-user confidence, practitioners also needed to be able to “bracket” their own feelings about VCs, even if these were negative [40,62,67].

#### Meso requirements

Some service-users did not have the requisite technology to access VCs and equipment was made available to them, for example, by loaning equipment [27,28,30,31,38,42,45,47–50,53–55,62,64,65,68,74–77,82,84,86,87]. Hughes [49] documents practical challenges arising from such arrangements, in relation to a telecare service for colorectal patients in an English hospital. Five tablets were loaned to patients for VCs, funded by a local stoma support group. Before doing so, the hospital’s IT department installed security measures and virus protection, locked the tablets so patients could use them only to access hospital information and created Skype accounts for each tablet. As only 5 tablets were available, they were prioritised for use by stoma patients. Other colorectal patients had to use their own devices. The authors describe challenges in obtaining funding for tablets and establishing legal responsibility if they were damaged while with patients (still unresolved at the time of writing the paper). This suggests that navigating financial, logistical, and legal implications within organisations is likely to be a further requirement, if equipment is loaned to service-users.

Some organisations provided access through “hub and spoke” models [26,58,60,83], where service-users, often from rural or deprived areas, travelled from home to a “spoke” facility, such as a local health centre, and used the technology there to consult with practitioners in distant “hubs.” In other cases, service-users were located in research or healthcare accommodation and used the equipment there [23,25,34,36,57,63]. In these contexts, there was a requirement for backup planning and keeping spares to replace lost equipment [60].

In response to service-users’ preference for direct contact with practitioners, some studies offered hybrid or blended provision, which incorporated at least some face-to-face interaction [27,31,34,38,43,47,62,68,71,77,87]. Several authors discussed the requirement for such provision; Christensen and colleagues [38], for example, noted that most patients and practitioners in an intervention for older people with depression expressed a need for face-to-face contact before VCs took place to establish the relationship and most preferred blended or hybrid models of provision, which they thought helped to maintain warm personal contact with nurses.

Organisations needed to plan VC services carefully, taking into account financial, logistical and workflow issues and multidisciplinary perspectives, including those of different practitioners, service-users, and IT professionals [27,32,39,48,49,56,61,68,79]. The importance of resource and workload planning was emphasised, ensuring staff had time to develop and support VCs [32,47,79,82] and facilitating inter-professional communication when practitioners from different disciplines or departments were involved [26]. Organisations needed to select the most appropriate equipment or service models^68^ and VC platforms to meet service-user and practitioner needs [29,80], which might involve conducting feasibility studies [68]. Team-building and staff development for VC services were required, in which managers reviewed and addressed practitioners’ needs, training and support [38] and facilitated team bonding [60].

To facilitate VC services and embed them within wider provision, authors recommended integrating VCs within organisational IT systems such as patient records systems or portals [43,61,71]. For example, in a study about the remote management of asthma, service-users recorded their symptoms on a patient portal using their smartphones [61]. These symptoms were then available to nurses to discuss with patients in VCs. Nurses believed this integration provided them with timely, relevant clinical information, enabling them to offer a better service: “*the information would be documented in the TEAMS note*, *and the patients were actually able to tell me the information*, *which is huge*… *I got more information*, *what their peak flow was*, *objective information”* (p. 7). A need for organisations to assure security was highlighted, for example, by choosing software that complied with national and international data security legislation [25,46,47,68].

#### Macro requirements

**Fig 3:**
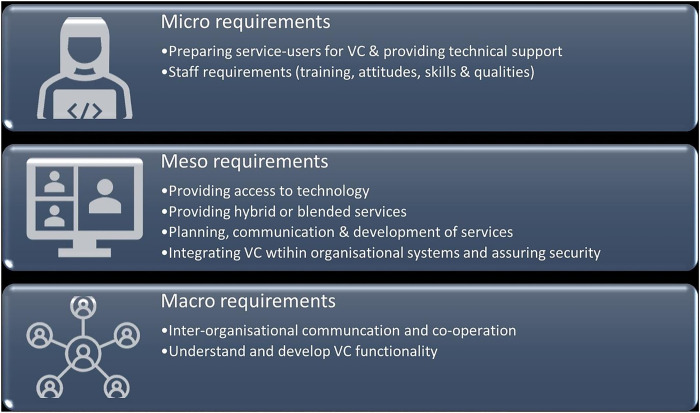
Micro, meso, and macro requirements for VC about here.

Some VC services involved practitioners from different organisations [26,30,32,58,60,83], requiring interorganisational communication and cooperation [30,32,60]. This was particularly important in “hub and spoke” models [26,58,60,83]. A need for further development of VC functionality at regional or national levels was also identified [24,25,27,30,39,41,66,68,79], as was the requirement for service providers to understand the characteristics of areas they serve, such as broadband capabilities or access to healthcare settings [30,37]. Fig 3 summarises micro, meso, and macro requirements for VCs.

## Discussion

VCs between nurses, allied health professionals, psychological therapists, and adult service-users are increasingly common, as the number of citations included in this review illustrate. Where VCs were compared with other modes of delivery, such as face-to-face, they were most often found to be comparable or better, and mode of delivery was a determining factor in whether some service-users received treatment at all. Where VCs were found to be less effective than other modes in comparative studies, reasons included practitioners’ and service-users’ lack of confidence in using the technology [53]; practical difficulties using VCs to carry out certain examinations or processes [23]; and service-users struggling to understand questions [36]. These factors were broadly reflected in other studies as reasons why some VCs were not experienced positively.

We identified a nuanced picture of service-users’ experiences of VCs, moving between feelings of connection to disconnection and ease of access to challenges to access. This reflects findings by other authors [10,14]. The review provided evidence that service-users’ personal attitudes and personalities (for example, whether they felt more comfortable on their own or in the company of others) affected whether they experienced VCs as engaging and empowering or not [44,57]. Kysely and colleagues [57] recommended further research into the effect of personality and other individual factors on clients’ expectations and willingness to engage in online therapy. Aspects of the technology itself could contribute to these feelings of connection, such as the use of collaborative chat and drawing functions [66,74], whereas limitations of the technology could have the opposite effect, such as poor internet connectivity or software incompatibility [27,73,74].

Impacts on service-users varied also, and sometimes contradicted assumptions, such as the assumption that older people may struggle to use technology. This review found that many service-users were excluded from VCs, with some studies excluding participants if they did not have capacity to consent to involvement or, in researchers’ or clinicians’ judgement, to participate in VC; if they could not speak or communicate in the main language(s) of the country in which the research was taking place; or if they did not have access to the required technology. Other authors have found that VC studies tend to focus on carefully selected service-users while neglecting others, which raises issues of digital exclusion, as well as questions about the applicability of VCs more widely [10,16]. In a systematic review of inequalities in general practice remote consultations, Parker and colleagues [2] recommended caution in extending the use of such consultations until the impact on clinical outcomes and quality of care for these neglected groups was known.

In response, we recommend those planning future VC services/studies consider eligibility criteria carefully to minimise exclusion. They may consider whether, if service-users do not have capacity to consent to a study, they could seek consent by proxy from families or practitioners and whether others, such as family members/caregivers, might support service-users’ involvement in VCs, for example, by helping them with technology. Likewise, providers and researchers could consider arrangements to include service-users who speak other languages by, for example, using interpreters, translation software, and/or the language skills of caregivers or practitioners in consultations. The ethical implications of such arrangements need to be considered carefully and built into services from the outset, prioritising patient safety.

This review identified micro, meso, and macro requirements for conducting and supporting VCs. These include, at the micro or individual level, providing clear orientation and ongoing technical support for service-users. In view of the wide-ranging experiences and expertise in providing support for VCs demonstrated in the review and to avoid providers “reinventing the wheel,” we recommend that, where possible, researchers share user guides and technical support documentation as supplementary materials for published papers and service-providers share such materials through organisational (and, ideally, cross-organisational) sites, such as best practice or innovation websites and hubs. These could complement existing publicly available resources [88–90].

The review also found practitioners need to develop and maintain technical and relational skills and qualities to help service-users feel connected and empowered during VCs and offer ease of access. We recommend both intra- and inter-professional continuing professional development to support practitioners in this work. Intra-professional development may be useful when developing discipline-specific skills, such as training physiotherapists how to support patient exercise online, whereas intra-professional work may help practitioners develop skills and qualities that support effective VCs across disciplines. These include demonstrating empathy online, establishing and maintaining trusting relationships, and helping users focus on consultations and minimise distractions. To supplement existing training [91,92], we believe it may be useful to find ways for psychological therapists to share expertise with other practitioners who offer VCs and vice versa, given the formers’ skills in developing therapeutic bonds with service-users online, demonstrated in this review. Psychotherapeutic methods have already been used in empathy training for medical students and might provide a further useful model for future research [93].

At meso or organisational levels, we identified requirements relating to service-users’ access to VC technology through equipment loan or service models such as hybrid/blended. Hybrid provision was common and offers some of the advantages of face-to-face consultations, such as immediate relational interaction, and of VCs, such as reducing travel. However, because it requires both organisational accommodation and remote technology, it may be expensive and complex to manage and requires careful planning and organisation. Addressing such challenges was a further requirement identified in the review, through measures such as planning and communication, integrating VCs within organisational IT systems and assuring secure services. Ethnographic research on VCs undertaken within the English National Health Service has highlighted similar needs [15].

Finally, at macro levels, requirements were identified to embed VC services within interorganisational processes through cooperation and by understanding and developing VC functionality. This is challenging and complex work, as the technology is changing constantly, and it is important organisations de-invest in outdated technology and look to streamline ways of working. Wherton and colleagues [15] make a similar point, noting VC development is often disrupted when attempting to link new technology with legacy systems and standards and when aligning administrative routines to new environments. They recommend an approach to adoption of VCs at scale which embeds both social/communicative and technical aspects, continually adjusting technology and work processes to become better aligned, and engaging with local and national decision-makers to ensure commissioning and regulatory structures facilitate this work.

### Limitations

This study did not receive external funding and was undertaken alongside other work priorities. Therefore, it could not be completed according to the rapid timescale specified in the protocol and instead took 2 years and searches were not updated beyond March 2021. If we had focused the review more narrowly, it may have been completed more quickly. Secondly, because only papers in English were included, research that reports experiences of people from a wider range of countries and ethnicities may have been missed. This is important, because non-Western/non-English-speaking populations may benefit particularly from VCs and researchers and practitioners in those countries, in turn, need evidence to support its development [5]. Future, funded reviews might address this point by including non-English texts to capture more diverse perspectives. Although some of our findings may be applicable in such countries, such as the need for practitioners to develop relational and technical skills to support VCs, we do not know whether all apply. Finally, the unfunded nature of the review meant that reviewers had capacity only to answer the research questions and not to develop detailed strategies to address issues raised. However, we hope our recommendations are helpful to researchers and service-providers in suggesting areas of focus as they develop VC provision in the future.

## Conclusion

In this review of 65 papers, we found different applications of VCs, conducted between nurses, allied health professionals, psychological therapists, and adult service-users, illustrating the global growth of VCs. However, many service-users were excluded from VCs.

### Recommendations for future research and service development

To address gaps or areas for development identified in this review, we recommend service-providers and researchers consider eligibility criteria carefully, to minimise exclusion from services and studies. To support the work of those developing VC programmes, we recommend sharing user guides and technical support documentation publicly and both intra- and inter-professional continuing professional development for practitioners, focusing on the technical and relational skills that service users value, such as the ability to convey empathy online.

## Supporting information

S1 Checklist PRISMA checklist. (DOCX)

S1 Data Study details table. (XLSX)

S1 File MMAT (quality assessment tool) for the review. (XLSX)
